# Simulating Pollutant Dispersion from Accidental Fires with a Focus on Source Characterization

**DOI:** 10.5696/2156-9614-11.30.210612

**Published:** 2021-06-17

**Authors:** Marzio Invernizzi, Francesca Tagliaferri, Selena Sironi, Gianni Tinarelli, Laura Capelli

**Affiliations:** 1 Politecnico di Milano, Department of Chemistry, Materials and Chemical Engineering “Giulio Natta”, Milan, Italy; 2 ARIANET, Milan, Italy

**Keywords:** atmospheric dispersion modeling, environmental impact, fire simulation, models comparison, sensitivity analysis

## Abstract

**Background.:**

Storage tanks in oil and gas processing facilities contain large volumes of flammable compounds. Once the fuel-air mixture is ignited, it may break out into a large fire or explosion. The growing interest in monitoring air quality and assessing health risks makes the evaluation of the consequences of a fire an important issue. Atmospheric dispersion models, which allow for simulation of the spatial distribution of pollutants, represent an increasingly widespread tool for this type of evaluations.

**Objectives.:**

The present study discusses the set up and results of a modeling study relevant to a hypothesized fire in an oil refinery.

**Methods.:**

After choosing the most suitable dispersion models, i.e. the Lagrangian model SPRAY and the puff model CALPUFF, estimation of the required input data is discussed, focusing on the source variables, which represent the most uncertain input data. The results of the simulations were compared to regulatory limits to effectively evaluate the environmental consequences. Finally, a sensitivity analysis was employed to identify the most influential variables.

**Results.:**

The simulation results revealed that ground concentration values were far below the cited long-term limits. However, the most interesting outcome is that depending on the dispersion model and the source type modeled, different results may be obtained. In addition, the sensitivity study indicates that the source area is the most critical variable, since it determines a significantly different behavior depending on the modeled source types, producing, in some cases, variability in the pollutant ground concentrations on selected receptors up to +/− 60%.

**Conclusions.:**

Depending on the selected model and the algorithms available to describe the physics of emission, the results showed a different sensitivity to the input variables. Although this can be explained from a mathematical point of view, the problem remains of choosing case by case the option that best approximates the real behavior of the incidental source under investigation.

**Competing Interests.:**

The authors declare no competing financial interests.

## Introduction

Storage tanks in oil refineries contain large volumes of flammable compounds. Once the fuel-air mixture is ignited, it may break out into a dangerous fire with devastating consequences.^1^,^2^

Atmospheric dispersion modeling represents an essential tool to assess the consequences of accidental fires by simulating the release of hazardous substances, thus quantifying the ground level concentrations of the emitted pollutants.

To perform a modeling study, input data related to the simulated domain and meteorological data are required. Therefore, it is important to have this data available and understand how to process data within the model. Furthermore, data concerning the emission scenarios are needed, defining the emitted pollutants, sources, and their location in the domain.

In the case of complex sources (e.g., accidental fire), the retrieval of representative emission data for dispersion modeling studies may be extremely problematic, as the source geometrical features are not directly measurable. Since multiple equipment types may be involved and due to the presence of flame, the source area cannot be directly estimated during an accidental fire. Thus, the geometrical features of the emission source need to be defined considering correlations available in the literature.

Due to the high uncertainty associated with the source term parameter estimation in case of fires,^3^ sensitivity analysis is strongly recommended at the end of a modeling study to identify the most influential variables.^4^

The present study explores the procedures to create a dispersion modeling study to evaluate the environmental consequences of a hypothesized fire in an oil and gas plant. For this purpose, the study discusses the choice of dispersion models to be used and estimation of the required input data, focusing on the source variables which represent the main criticalities for this type of simulation. The results of the simulations are presented in terms of ground concentrations of different pollutants. In order to make these results meaningful, the uncertainty associated with the input data is analyzed by examining the impact of possible errors in the source characterization. To quantify how much the environmental impact would change due to inappropriate characterization of the source, the simulation was repeated at the extreme values of the plausible ranges of variation for each variable. To perform the simulations, the Lagrangian particle model SPRAY^5^ and the Lagrangian Gaussian puff model CALPUFF^6^ were selected since they currently represent the most common tools to simulate pollutants dispersion from fires. 7–10

## Methods

The hypothesized case study regards an incidental fire in a petroleum refinery involving the finishing section of the gas oil desulfurization unit. In real cases the duration of a fire must be estimated based on the available documental material and the depositions of the people who were present. In the present hypothesis, the fire lasts three hours, after which the phenomenon is completely controlled, and occurs during a typical winter morning with cloud cover.

### Atmospheric dispersion models and suitable simulation tools

The model choice is based on the analysis of the scientific literature and technical legislation. Moreover, the specificities of the hypothesized case study need to be taken into account, such as the particular type of source (fire) and the large simulation domain. On one hand, the use of simple Gaussian models, which have a very fast response time, is not advisable in case of large simulation domains^11^ because they consider steady state conditions: they cannot adequately describe the dispersive phenomenon, since only one meteorological condition is not representative of the wind field variations on the entire domain. Conversely, Eulerian and fluid dynamics models are very advanced simulators, but at the same time they are complex and require a long computational time.

In addition, fluid dynamics models simulating fires are interesting, or even necessary, when the dispersion occurs in urban areas, where the influence of buildings on the dispersion is dominant or where the scale to consider is the so-called meteorological microscale (<1 km).^12^–^15^ However, this is not the case for the condition hypothesized in the present study.

There are several examples in the literature of studies carried out using puff models, and specifically CALPUFF, for the simulation of pollutant dispersions from fires.^7^,^8^ The authors justify the model choice because it can treat buoyant sources. There are not as many articles regarding the application of SPRAY for fires, presumably because it is a more recently developed model. On the other hand, there are some studies in which both CALPUFF and Lagrangian particle models are used.^16^,^17^ As an example, in the first paper, the choice of using non-steady state three-dimensional (3D) models to evaluate the impact from a fire in a waste storage plant is justified by the need to provide a 3D description of the meteorological field in order to account for some essential characteristics such as wind shear, variable emissions over time and buoyant area source.

Furthermore, the suitability of these models for the selected case study is supported by the analysis of the technical standardization currently available in Italy. The standards UNI EN 10796: 2000^18^ and UNI EN 10964: 2001^19^, which define the scenarios for the implementation of the different models suggesting the best model to be used for different situations, confirm the suitability of the selected tools to simulate fires.

In the end, for the present study, a puff model (i.e. CALPUFF) and a Lagrangian particle model (i.e. SPRAY) were chosen, since both comprise specific tools for modeling fires. As previously mentioned, the available scientific literature and technical legislation support this choice.

To perform the simulations, different algorithms available to describe the physics of emission are set in the models: with CALPUFF the fire is simulated as a buoyant area source according to the indications of the User's Guide,^6^ whereas with SPRAY the fire is simulated with two different approaches: as a point source (e.g. stack) and as a fire, characterized by 10% of the emitted particles with no buoyancy flux. In the last case, SPRAY considers the *smoldering* phase of a fire, where “smoldering is a slow, low-temperature, flameless form of combustion, sustained by the heat evolved when oxygen directly attacks the surface of a condensed-phase fuel”.^20^ Thus, the fire is simulated taking into account a cold fraction of pollutant, which immediately falls to the ground, without being dragged into the plume rise.

### Steps required to set the simulation

Before running the simulations, some preliminary evaluations were needed to define all the input data the model requires. The definition of the simulation domain, in which the environmental impact has to be assessed, is of primary importance. To select a suitable area, weather data showing the plume direction should be taken into account. For the hypothesized case study, a rectangular domain of 25×25 km was identified with a mesh grid of 250 m. Then, assuming a plume evolving in a southwestern direction, the source was located at the northeastern corner of the domain. In addition, the domain extension should be chosen to include any region of sensitive receptors such as residential areas or buildings of public interest. Thus, for a more precise analysis, some discrete receptors were located to be representative of possible places of interest.

The second point involves characterization of the terrain, by extracting the orography and the land use from a reference database. Furthermore, a meteorological domain needs to be identified. The meteorological data necessary for the simulation are 3D prognostic Weather Research and Forecasting (WRF) data purchased from Lakes Environmental (https://www.weblakes.com). Then, each model processes the WRF data using the specific meteorological tools (i.e. SWIFT for SPRAY and CALMET for CALPUFF), which are diagnostic “mass consistent” models for complex terrain. They generate 3D wind fields inside the meteorological domain, which has been set equal to the computational grid.

### Source term definition

To complete the set of input data required by the models, it is necessary to fully characterize the source term. This characterization includes the definition of the geometry of the emission source as well as its physical-chemical variables. The emission scenario shall be defined by identifying the amount of fuel burnt and the pollutants released with their emission factors. Thus, starting from the evaluation of the amount of fuel, mass balances are considered. Based on the knowledge of the mass flow rates of the species inside the involved equipment and the release time, it is possible to estimate this quantity.

Then, for an incidental fire, a fundamental point is the definition of the species to be simulated, and the quantities emitted. Based on a search of the scientific and technical literature, it is possible to assume that the most significant pollutants released during gas oil combustion are carbon dioxide (CO_2_), carbon monoxide (CO), generic unburned hydrocarbons (CH), particulate matter (PM), sulfur oxides (SO_X_) and nitrogen oxides (NO_X_).^21^,^22^

The Society of Fire Protection Engineers (SFPE) Handbook of Fire Protection Engineering^23^ was chosen for the estimation of emission factors, as it is considered the most authoritative and reliable source. The document reports the emission factors for CO_2_, CO, CH, and PM for different fuels. Among all the listed species, the generic “hydrocarbon” and kerosene, which represent the most similar fuels to the one hypothesized for the simulate fire, were considered to select the emission factors. However, since the SFPE Handbook of Fire Protection Engineering does not consider NO_X_ emissions, the AP-42 of the USEPA^24^ was used to estimate the emissions of this species.

The emission factor for SO_X_ was estimated assuming the complete (stoichiometric) transformation of the elemental sulfur (S) in the original fuel, hypothesized equal to 100 ppm, to sulfur dioxide (SO_2_).

At this point it is possible to estimate the emission rate for each compound as the product of its emission factor and the amount of fuel burned, divided by the event duration. Furthermore, the model requires the geometrical features of the simulated source. After evaluation of the source diameter from surveys and analysis of the existing documentation, it is necessary to characterize the total height, defined as the sum of the involved equipment and the flame height (H_f_) given by the Heskestad correlation *(Equation 1)*^23^*:*

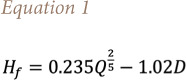
where *Q* is the heat release (kW) and *D* is the source diameter (m).

The Heskestad formula requires the estimation of the heat release rate that may be derived from the Babrauskas correlation *(Equation 2)*^23^*:*

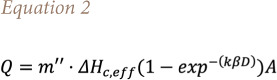
where ΔH_c,eff_ is the net heat of combustion, kβ an empirical constant, A is the source area, and m” is the specific mass burning rate (kg m^−2^ s^−1^).

Knowing the heat release it is possible to derive the fire smoke rise velocity *(Equation 3)*^25^*:*

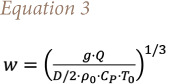
where T_0_ is the ambient temperature, ρ_0_ the air density, cp the specific heat of air at constant pressure, g = 9.81 [m/s^2^], Q the heat release and D the source diameter.

Finally, to define the fire temperature, the hydrocarbon fire curve, reported in EN 1363-2:1999^26^ showing the trend of the temperature as a function of time, was considered. Thus, the maximum achievable temperature of about 1100°C was used for the hypothesized case study since it is rapidly achieved after a few minutes.

To characterize the source from a geometrical point of view, the definition of the diameter and the height is insufficient. Indeed, each model requires a specific variable to run the simulations. As far as CALPUFF is concerned, the initial vertical dispersion coefficient σ_z0_ enables definition of the initial dimension of the puff in the vertical direction. Concerning the base-case scenario, σ_z0_ is evaluated according to the following formula, as suggested in literature for elevated source for Gaussian plume model *(Equation 4)*^27^*:*

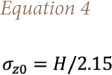
Where *H* is the source height.

A different parameter, but conceptually very similar to σ_z0_, is implemented in the SPRAY model. The Lagrangian particles model requires the definition of the vertical dimension of the “emission parallelepiped”, Δz. SPRAY generates particles uniformly distributed on a “terrain following” parallelepiped centered in P (X_0_, Y_0_, Z_0_), which are the coordinates of the emission region center of gravity, whose vertical dimension is Δz. In other words, this parallelepiped can be thought of as a box in which the particles initially appear. Thus, they are released in a vertical region ranging from Z_0_− Δz/2 and Z_0_ + Δz/2, with Z_0_ possibly coincident with the source height. Therefore, this variable represents the vertical dimension for the release of the particles: it is a parameter describing the initial condition of the emission and it should be defined to reproduce the geometrical features of the emission region as well as possible. The geometrical features of the emission region refers not only to the effective source dimensions, but also to the dynamic effects influencing the emission. For instance, due to the configuration of the stack or possible adjacent building, the plume may not rise freely in the atmosphere: some aerodynamic effects due to the way the wind moves around adjacent buildings and the stack can force the plume toward the ground instead of allowing it to rise. In case of stack tip downwash, the plume is drawn downward behind the stack and the pollutant dispersion is reduced. As the air moves over and around buildings, turbulent wakes are formed: depending on the stack height, it may be possible for the plume to be pulled down into this wake area (building downwash) resulting in high concentrations immediately downwind of the source. Therefore, to reproduce the emission region as completely as possible, a conservative value equal to twice the source height was identified.

On the basis of these considerations, the characteristic variables of the emission source are shown in Table 1.

**Table 1:** Input Variables Relevant to the Emission Source

**A (m^2^)**	**T (K)**	**H (m)**	**Quantity (ton)**	**v (m/s)**	**Δz SPRAY (m)**	**σ_z0_ – CALPUFF (m)**	**PM (g/s)**	**CO (g/s)**	**CO_2_ (g/s)**	**NO_x_ (g/s)**	**SO_2_ (g/s)**	**HC (g/s)**
20	1373	15	11.2	8.16	30	6.98	51.9	20.7	2800	10.4	0.2	7.3

Abbreviations: A, source area; Δz, vertical dimension of the emission parallelepiped; H, source height; HC, generic unburned hydrocarbons; σz0, initial vertical dispersion coefficient; PM, particulate matter; T, emission temperature; v effluent exit velocity

### Sensitivity analysis for source term variables

The high uncertainties associated with the characterization of the source term in case of fire is a key issue.

These variables are highly uncertain due to the impossibility of direct measurement from the accident site. In addition, the choice of σ_z0_ e Δz represents a critical point since clear indications are usually not available and their definition is left to the professional judgment of the modelist. For this reason, it is important to account for a plausible range of variation for the selected variables and to investigate what happens when simulating not only the reference value of the base-case *(Table 1),* but also the extreme values of the range.

Thus, to make the simulation results meaningful, a sensitivity analysis is strongly recommended. The second part of the study discusses the impact on the model output of possible errors in the input data related to the emission source.

To perform the modeling study, the previously identified scenario *(Table 1)* was used as a reference for comparison with the other emission scenarios that are defined in order to evaluate the models' sensitivity to the source input variables. Thus, starting from the “base-case”, it was decided to investigate alternative emission scenarios, shown in Table 2, by changing one of the variables (in bold) relevant to the emission source.

**Table 2:** Input Variables of the Investigated Alternative Emission Scenarios

**Scenario**	**A (m^2^)**	**T (K)**	**H (m)**	**Quantity (ton)**	**V (m s^−1^)**	**PM (mg m ^−3^)**	**Δz − SPRAY (m)**	**σ_z0_ − CALPUFF (m)**
A1	**100**	1373	15	11.2	6.21	51.852	30	6.98
A2	**10**	1373	15	11.2	9.17	51.852	30	6.98
H1	20	1373	**20**	11.2	8.16	51.852	30	6.98
T1	20	**1273**	15	11.2	8.16	51.852	30	6.98
T2	20	**1473**	15	11.2	8.16	51.852	30	6.98
Q2	20	1373	15	**22.4**	10.28	103.703	30	6.98
Q2A1	**100**	1373	15	**22.4**	7.86	103.703	30	6.98
Q5A1	**100**	1373	15	**56.0**	10.68	259.260	30	6.98
Δz2	20	1373	15	11.2	8.16	51.852	**15**	-
Δz3	20	1373	15	11.2	8.16	51.852	**20**	-
Δz4	20	1373	15	11.2	8.16	51.852	**25**	-
σ_z0,1_	20	1373	15	11.2	8.16	51.852	**-**	**3.49**

Abbreviations: A1/A2, alternative scenarios for source area; Δz2/Δz3/Δz4, alternative scenarios for vertical dimension of the emission parallelepiped; H1, alternative scenario for source height; σz0,1, alternative scenario for initial vertical dispersion coefficient; Q2, alternative scenario for amount of fuel; Q2A1/Q5A1, alternative scenarios for source area and amount of fuel; T1/T2, alternative scenarios for emission temperature

**Bold** indicates variables changed in the alternative scenarios with respect to the reference base-case.

Among all the selected species, PM was chosen as the target species for the investigations, since it is the pollutant whose emission is considered to be most critical in the case of incidental fires. The other emitted compounds were not simulated in the alternative scenarios.

Obviously, scenarios in which the vertical dimension of the emission parallelepiped (Δz) has been changed are specifically referred to the Lagrangian particle model. On the other hand, the alternative case referred to the σ_z0_ has been only developed in the CALPUFF model. It is worth underlining that, when using SPRAY—fire model, scenarios T1 and T2 were not investigated, since the fire temperature is not an input variable required by the software to simulate this type of emission source.

## Results

At the end of the simulations it is possible to process the results to extrapolate ground level concentration maps representing the pollutant concentration within the simulation domain. Specifically, in order to simulate the worst-case condition, the maximum 1-hour concentrations for the different species were computed and presented in the figures below. It is worth noting that for CALPUFF— buoyant area and SPRAY—fire, the scale of the concentration values reported in the maps is the same.

The only pollutant whose concentration reached values comparable with the reference limits of air quality, as discussed below, is PM. Thus, PM was chosen as a reference species for any other investigation, whereas the other species were excluded from further evaluations.

From the concentration maps previously shown, it is evident that the different source types modeled with SPRAY (i.e. point vs fire) lead to different concentrations values, especially in the vicinity of the emission source. To explore this different behavior, some receptors placed along the plume axis at different distances from the source were considered *(Figure 7).*

**Figure 1:**
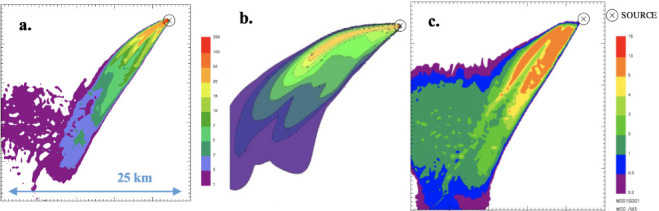
Maximum ground level concentration maps of particulate matter resulting from SPRAY-fire (a.), CALPUFF- buoyant area (b.), and SPRAY-point (c.)

**Figure 2:**
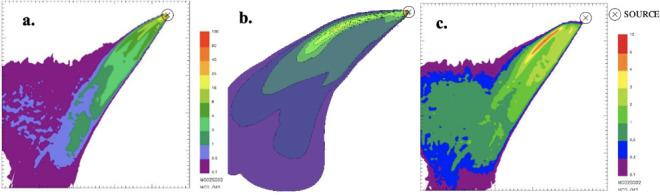
Maximum ground level concentration maps of carbon monoxide resulting from SPRAY-fire (a.), CALPUFF- buoyant area (b.), and SPRAY-point (c.)

**Figure 3:**
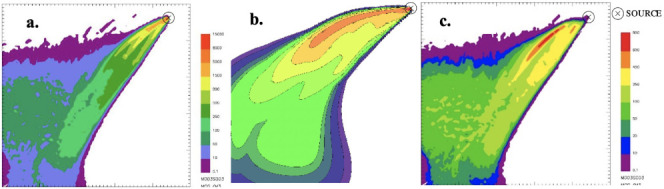
Maximum ground level concentration maps of carbon dioxide resulting from SPRAY-fire (a.), CALPUFF- buoyant area (b.), and SPRAY-point (c.)

**Figure 4:**
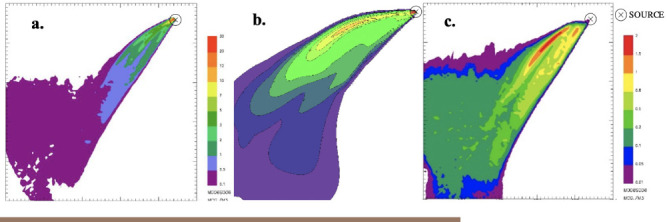
Maximum ground level concentration maps of generic unburned hydrocarbons resulting from SPRAY-fire (a.), CALPUFF- buoyant area (b.), and SPRAY-point (c.)

**Figure 5:**
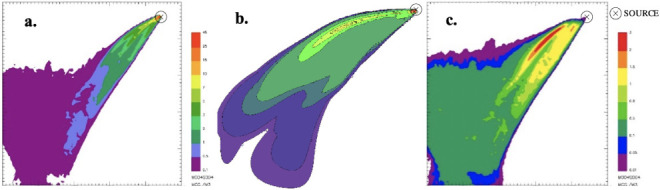
Maximum ground level concentration maps of nitrogen oxides resulting from SPRAY-fire (a.), CALPUFF- buoyant area (b.), and SPRAY-point (c.)

**Figure 6:**
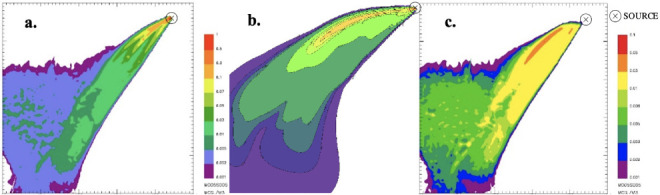
Maximum ground level concentration maps of sulfur dioxide resulting from SPRAY-fire (a.), CALPUFF- buoyant area (b.), and SPRAY-point (c.)

Furthermore, to integrate the information provided by the maps (*Figure 1–6*), the maximum PM concentration was computed by the model on a set of selected discrete receptors, representative of “sensitive points” in which the assessment of environmental impact is particularly interesting. These results are reported in Table 3.

**Figure 7—:**
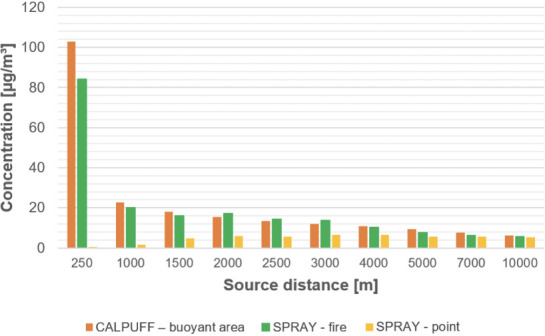
Maximum particulate matter concentrations on the selected receptors for the different combinations of dispersion models and source types

**Table 3:** Maximum Particulate Matter Concentration Values at Selected Receptors Calculated by CALPUFF (left) and SPRAY (right) Models

**CALPUFF**		**SPRAY**		
Receptor	**Concentration (buoyant area) (μg/m^3^)**	**Receptor**	**Concentration (point source) (μg/m^3^)**	**Concentration (fire) (μg/m^3^)**
**1**	114.48	1	12.97	213.2
**2**	1.05	2	4.19	4.99
**3**	14.69	3	4.28	23.6
**4**	11.46	4	5.67	19.45
**5**	18.83	5	5.60	22.87
**6**	19.29	6	6.64	23.31

It is important to highlight that the selected receptors are not necessarily the same for CALPUFF and SPRAY, even though they have been chosen by the same logic (for instance, the discrete receptors identified as 1 in Table 3 correspond to the receptors where the maximum concentration has been calculated by the two models). This is because, for instance, at a point in which SPRAY computes a non-zero concentration value, CALPUFF may produce null results (or vice-versa), due to the different plume directions.

The results of the different models discussed in this section are strongly affected by the choice of the input data, which have an intrinsic uncertainty. Thus, it is advisable to quantify the output variation provided by an input change, thereby performing a sensitivity study. In particular, for an accidental fire, the input variables relevant to the source characterization are the most uncertain. Therefore, the results of a sensitivity analysis applied to the source term variables is presented below.

### Sensitivity analysis for source variables

To determine how the results of the simulations are affected by a perturbation of the input variables, the variability (%) of the maximum PM concentration was computed by making the difference between the concentration resulting from the alternative scenarios and the one of the base-case, as shown by Equation 5:


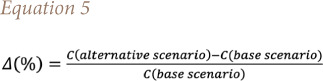


Tables 4–6 report the variability (%) computed on the above mentioned receptors for the different scenarios and source types. The percentage variations are shaded in green if lower than zero with increasing intensity as further away from zero. Red shading is used for positive values.

**Table 4:** Percent Variation of Particulate Matter Concentration at the Selected Receptors Resulting from the Simulations of Alternative Scenarios Compared to the Base-case for SPRAY (Point)

**SPRAY (point source)**											

**Receptor**	**A1**	**A2**	**H1**	**T1**	**T2**	**Q2**	**Q2A1**	**Q5A1**	**ΔZ2**	**ΔZ3**	**ΔZ4**
1	−52%	48%	2%	1%	−4%	70%	−5%	105%	8%	4%	5%
2	−25%	8%	0%	−5%	2%	90%	51%	237%	5%	−6%	3%
3	−65%	51%	14%	1%	−1%	74%	−39%	19%	10%	6%	3%
4	−39%	25%	11%	6%	3%	89%	12%	141%	10%	9%	7%
5	−39%	23%	3%	2%	−2%	91%	10%	149%	8%	1%	4%
6	−60%	50%	17%	2%	0%	81%	−31%	40%	14%	9%	3%

**Table 5:** Percent Variation of Particulate Matter Concentration at the Selected Receptors Resulting from the Simulations of Alternative Scenarios Compared to the Base-case for SPRAY (Fire)

**SPRAY (fire)**									

**Receptor**	**A1**	**A2**	**H1**	**Q2**	**Q2A1**	**Q5A1**	**ΔZ2**	**ΔZ3**	**ΔZ4**
1	10%	−1%	−22%	86%	97%	355%	−7%	−3%	−1%
2	2%	−2%	3%	64%	65%	263%	2%	3%	−2%
3	3%	0%	0%	96%	100%	392%	3%	1%	2%
4	2%	1%	2%	95%	101%	388%	3%	1%	2%
5	1%	0%	0%	95%	102%	387%	1%	0%	0%
6	1%	0%	−1%	91%	95%	369%	0%	1%	1%

**Table 6:** Percent Variation of Particulate Matter Concentration at the Receptors Resulting from the Simulations of Alternative Scenarios Compared to the Base-case for SPRAY (Buoyant Area)

CALPUFF (buoyant area)									

**Receptor**	**A1**	**A2**	**H1**	**T1**	**T2**	**Q2**	**Q2A1**	**Q5A1**	**σ_z0.1_**
1	−48%	42%	−17%	1%	0%	77%	−7%	100%	−10%
2	−62%	8%	−1%	1%	−4%	85%	−35%	43%	0%
3	−41%	36%	−5%	1%	−1%	71%	6%	131%	1%
4	33%	−53%	4%	−2%	1%	146%	141%	417%	−1%
5	−67%	74%	−7%	2%	−1%	39%	−40%	32%	1%
6	−46%	21%	−3%	1%	0%	83%	−4%	108%	−2%

## Discussion

To evaluate the effects of an accidental fire on public health, modeled concentrations need to be compared with the regulatory limits for air quality. For this purpose, the only regulatory reference currently available in Italy regarding air quality is the D. Lgs. 155/2010^28^ which prescribes the average values not to be exceeded over a long period (usually one year). Therefore, these regulatory limits are not directly comparable with the results of the present study, which instead is based on a short-term event and considers maximum hourly concentration values.

In the hypothesized scenario, for all source types, ground concentration values were far below the cited long-term limits. Indeed, the simulated accident is not a catastrophic event, but has been hypothesized as a limited event involving a single piece of equipment. The only pollutant whose concentration reaches values comparable with the reference limits of air quality (50 μg m^−3^) is PM. Thus, as mentioned in the previous paragraph, PM was chosen as reference species for any other investigation.

From the concentration maps shown in Figures 1–6, it is evident that different plume directions may be observed, depending on the dispersion model used. Indeed, starting from the same meteorological input data, the different meteorological tools (i.e. CALMET for CALPUFF and SWIFT for SPRAY) process the results in a slightly different way. In addition, comparing the different source types modelled with SPRAY (i.e. point versus fire), different concentrations values, especially in the vicinity of the emission source, are calculated by the model, as confirmed by the graph in Figure 7. This is attributable to the different algorithms implemented into the software to model the different sources. In particular, the models consider different plume rise computations for point sources and for fire/buoyant area sources. According to the SPRAY model for fires, which considers the fact that combustion in fires is incomplete, there is a cold fraction of particles that remain unburnt and immediately fall to the ground, without being dragged into the plume rise. This results in higher PM concentrations, closer to the source. In CALPUFF the buoyant area source model considers radiative heat losses due to the high plume temperature near the burning source. Consequently, the heat flux carried out by the plume along its trajectory will be reduced, leading to a lower buoyancy flux. On the contrary, for point sources, the maximized plume rise leads to very low concentration values close to the emission point *(Figure 7).* At a greater distance from the source (>5000 m), the maximum PM concentrations computed by the different models tend to become very similar, giving concentrations ranging from 5 to 10 μg m^−3^.

This is consistent with the results shown in Table 3, where the concentration values identified by the SPRAY–fire model and CALPUFF - buoyant area source at the selected receptors are higher than those obtained considering the point source, except for receptor 2 where very similar values were observed. Indeed, receptor 2 is the only one, of all the investigated points, located very distant from the source.

### Sensitivity analysis to source parameters

The results of the alternative scenarios enable one to immediately identify the most influential variables. One of these variables is the area of the source, which is also particularly interesting because of its different behavior depending on the source type considered. In particular, it significantly affects the model result when using the CALPUFF—buoyant area source model or the SPRAY— point source model, but leads to very low variations for the SPRAY model in combination with the specific fire option giving a maximum variability, at the selected receptors, of 10% *(Tables 4–6).*

Conversely, for CALPUFF and the point source simulated by SPRAY, the source area represents a highly influential variable since the simulations conducted by reducing the area from 20 m^2^ to 10 m^2^ (scenario A2) generally result in an increase of the simulated maximum PM concentrations of about 50%, whereas an opposite effect is obtained by increasing the area from 20 m^2^ to 100 m^2^ (scenario A1), generally giving decreased concentrations of about 60% *(Tables 4–6).*

This different effect may be justified from a mathematical point of view. Indeed, when using the SPRAY–point source model, the area influence can be explained by considering that the buoyancy flux computation is performed according to the Briggs equation^5^, which is proportional to the square of the source radius *(Equation 6):*


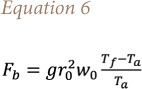


Here, an increase in area means a small decrease in velocity, whereas the source radius increases more significantly. Therefore, the dominant term is the second one, leading to an increased buoyancy flux and a reduction in ground level concentrations.

When using the CALPUFF–buoyant area source model, the radiative heat loss from the plume to the ambient air can be estimated through Equation 7^6^:


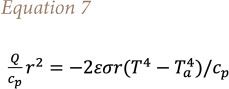


Here, an increase in radius implies a reduction of heat losses. Consequently, the plume rise increases and the pollutant concentrations decrease. On the other hand, if the SPRAY model is used in combination with the specific fire option, the influence of the area of the source on the model outputs turns out to be negligible. From a mathematical point of view, this can be explained from Equation 8, which is used for the calculation of buoyancy flux, in which neither velocity nor radius appear, giving that the buoyancy calculation is not affected by the source area^5^:


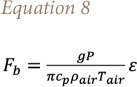


The second investigated variable is the source height, whose influence on the model output is less relevant. For all the investigated sources, the variability was of the same order of magnitude and it does not significantly affect the model results *(Tables 4–6).* The same consideration applies to the model's sensitivity to temperature. Its influence on the concentration values is even lower than those of the source height, giving a maximum variation on the selected receptors of 5% *(Tables 4–6),* indicative of an almost negligible contribution.

Considering the Q2 scenario, in which the amount of fuel burned is the double of those simulated in the reference scenario, it turns out that the concentration at the receptors is not exactly doubled. Indeed, the calculated variability is always lower than 100% (except for CALPUFF in receptor 4). The reason is that at constant area, increasing the fuel burned, the heat release by the fire rises, leading to an increase of the velocity (according to the Ingason equation, Equation 3) that promotes pollutant dispersion.

For the alternative scenarios in which the source area and the amount of fuel burned have both been modified (Q2A1 and Q5A1), the variabilities (%) generated by SPRAY–fire are significantly higher than those obtained with the other source types. This is because, when using the fire model, since the area is irrelevant, the variation in the PM concentration is due to the increase in the amount of fuel burned. Conversely, the other investigated sources are strongly affected by area variation. Thus, the high concentrations promoted by an increase in fuel burned are partly offset by a higher source area that generally reduces the concentration.

Furthermore, for CALPUFF, an additional scenario, in which the σ_Z0_ was halved compared to the base-case was investigated. Looking at the variability shown in Table 6, the initial vertical dispersion coefficient does not significantly affect the ground level concentration because, when halving the σ_Z0,_ the model result is subjected to a maximum variation of 9.5% in the point of maximum concentration, whereas for the other receptors this variability is below 2%.

As far as SPRAY is concerned, different scenarios have been identified to discuss the influence of Δz, whose estimation is not trivial. A first consideration concerns the percentage variations provided by the investigated input datum. Changing the vertical dimension of the “emission parallelepiped”, a significant output variability was not observed because, in almost all the detected receptors, it was lower than 10% *(Tables 4 and 5).*

In addition, comparing the two source types, their response to the input variation appears quite different. For the three investigated scenarios and in all the detected receptors, SPRAY–point source always showed a higher variation than the fire model. Thus, this behavior does not seem to be attributable to the position of the selected receptors, but it is a general feature resulting from the different way of modeling the source. Indeed, a change in the parameter Δz, which is representative of the vertical dimension of a “box” in which the particles initially appear, means a change in the dimension of the region from which the particles start to rise up due to the buoyancy. Thus, the plume rise is affected by this variable in the sense that the “idealized” plume containing the particles has a different initial shape and dimension according to this variable. Therefore, the fire model, considering a percentage of the emitted particles with no buoyancy flux, was less influenced by Δz.

## Conclusions

Atmospheric dispersion models represent an increasingly widespread tool to assess the environmental and health consequences of accidental events involving the release of hazardous pollutants. The present study details the set up and results of a modeling study relevant to a hypothesized fire in an oil and gas plant. To carry out this analysis, the Lagrangian model SPRAY and the puff model CALPUFF were selected.

In the first part of the study, the definition of the input data required for the simulations was presented. The characterization of the emission source was explained in detail since the estimation of the source term variables represents a critical issue in the case of accidental fires. From the results of the maximum ground level concentrations of the most significant species, the most relevant outcome is that depending on the dispersion model and the source type implemented, different results are obtained. However, in all cases, the simulated ground concentration values were below the corresponding regulatory limits for air quality.

Due to the high complexity associated with the source characterization for an accidental fire, a sensitivity analysis is recommended to evaluate the significance of results. The modeling study coupled with a sensitivity analysis allows identification of the most influential variables, focusing efforts on improved variable characterization, so as to reduce the total uncertainty.

The results of the present study indicate that the geometrical area of the emission source represents one of the variables that most significantly affects the model outputs. By considering the extreme values of the defined uncertainty range (10 m^2^–100 m^2^), the pollutant concentrations on some receptors vary up to +/− 60% when adopting the point source modeled by SPRAY or the CALPUFF buoyant area source. On the other hand, if the SPRAY model is applied with the specific fire source option, then the modeled concentrations result almost independently from this variable. The other variable that produced a high variability in the results is the amount of fuel burned. The simulations conducted doubling this variable led to simulated concentrations approximately doubled when the amount of fuel is increased by a factor of two.

While these two variables are those that led to greater variability, they are also the variables that have been varied most (except for σ_Z0_ which was halved). Nevertheless, the amount of fuel can be easily quantified through mathematical calculations and, therefore, its estimation is not a critical issue. On the other hand, the definition of the source area is more critical, thus care should be taken when setting this variable.

Furthermore, area is the only variable that determined a significantly different behavior when comparing SPRAY and CALPUFF. In particular, when adopting the options specifically recommended to simulate fires (i.e. buoyant area source for CALPUFF and fire model for SPRAY) the results showed a different sensitivity to the area. Although this can be explained from a mathematical point of view, the problem remains of choosing case by case the option that best approximates the real behavior of the incidental source under investigation. To do this, a model validation technique should be considered. In this way, it would be possible to evaluate the model capability to effectively predict the experimental observations, and thus assess the goodness of the model and eventually improve and optimize its performance.
